# The Role of EZH2 in Malignant Pleural Mesothelioma and Beyond: Current Practice and Future Perspectives

**DOI:** 10.1007/s11912-026-01754-x

**Published:** 2026-06-08

**Authors:** Mehmet Kucukoner, Tarik Demir, Abduljalil Mohammed Alsubaie, Ulas Alabalik, Aisha K Ibrahim, Mariaelena Pierobon, Lance Liotta

**Affiliations:** 1 Department of Medical Oncology, Dicle University Faculty of Medicine, Diyarbakır, Turkey; 2 The Ohio State University Wexner Medical Center, Columbus, OH USA; 3 Center for Applied Proteomics and Molecular Medicine, George Mason University, VA, USA; 4 Department of Pathology, Dicle University Faculty of Medicine, Diyarbakir, Turkey

**Keywords:** EZH2, Epigenetics, Mesothelioma, Immunotherapy, PRC2, BAP1

## Abstract

**Purpose of Review:**

Malignant pleural mesothelioma (MPM) remains a rare but highly aggressive malignancy with limited treatment options and poor prognosis. For nearly twenty years, platinum–pemetrexed chemotherapy has persisted as the unchanged standard treatment; although recent progress in immunotherapy has modestly disrupted this therapeutic plateau, survival outcomes remain disappointingly limited. This review aims to provide a comprehensive overview of the epigenetic landscape of MPM, focusing particularly on the oncogenic and therapeutic implications of enhancer of zeste homolog 2 (EZH2), and to discuss its potential as a target for novel therapeutic strategies and combination regimens.

**Recent Findings:**

Epigenetic dysregulation has emerged as a central driver of mesothelioma pathogenesis. EZH2, the catalytic component of the polycomb repressive complex 2 (PRC2), mediates histone H3K27 trimethylation, silencing tumor suppressor genes and promoting malignant transformation. In addition to its canonical role, EZH2 has non-canonical oncogenic effects that modulate transcription, apoptosis, DNA repair, and immune evasion. High EZH2 expression correlates with BAP1 loss, which enhances chromatin remodeling defects and disease aggressiveness. Preclinical and early clinical data demonstrate that EZH2 inhibitors—including tazemetostat, valemetostat, GSK126, EPZ011989, tulmimetostat, and novel PROTAC-based degraders such as MS1943—can suppress tumor progression, modulate the tumor immune microenvironment, and restore therapeutic sensitivity. Furthermore, combination approaches integrating EZH2 inhibition with chemotherapy or immune checkpoint blockade show synergistic potential in overcoming resistance.

**Summary:**

EZH2 represents a pivotal epigenetic regulator and a promising therapeutic target in MPM. Further understanding the dual canonical and non-canonical roles of EZH2 in tumor biology will be key to optimizing targeted and combinatorial treatment strategies. Future research should focus on translating EZH2 inhibition into clinical benefit, identifying predictive biomarkers of response, and exploring rational combinations with chemotherapy, targeted drugs, or immunotherapy to improve survival outcomes in mesothelioma patients.

## Introduction

Malignant Pleural Mesothelioma (MPM) is a rare and very aggressive form of cancer that develops from mesothelial cells in the pleural lining of the lung associated with asbestos exposure. The median overall survival is about 1 year, and a cure is very rare [1, 2]. In the standard first-line treatment of MPM, there were no new approved treatments other than pemetrexed and cisplatin chemotherapy [3]. In recent years, immunotherapy, an important field of ​​cancer treatment, namely immune checkpoint inhibitor drugs, has also been investigated in mesothelioma. In these studies, the combination of ipilimumab and nivolumab or the combination of pembrolizumab added to standard platinum-pemetrexed chemotherapy has shown an improvement in overall survival [4, 5]. With these studies, a new drug group other than chemotherapy has been approved for the first time in the last 20 years. Despite these new options, the prognosis of MPM is still very poor, with an average overall survival of not more than 14 to18 months, and a significant proportion of patients are resistant to chemotherapy and immunotherapy [6]. There is an unmet need for mesothelioma treatment and new pharmacological targets are needed. Along with cell cycle regulation, growth factors, angiogenesis and apoptosis, other important mechanisms in tumor biology such as epigenetic modifications and DNA damage repair have been investigated, especially in recent years [7]. However, despite promising preclinical studies, there is still no clinically approved targeted drug therapy.

**Fig. 1:**
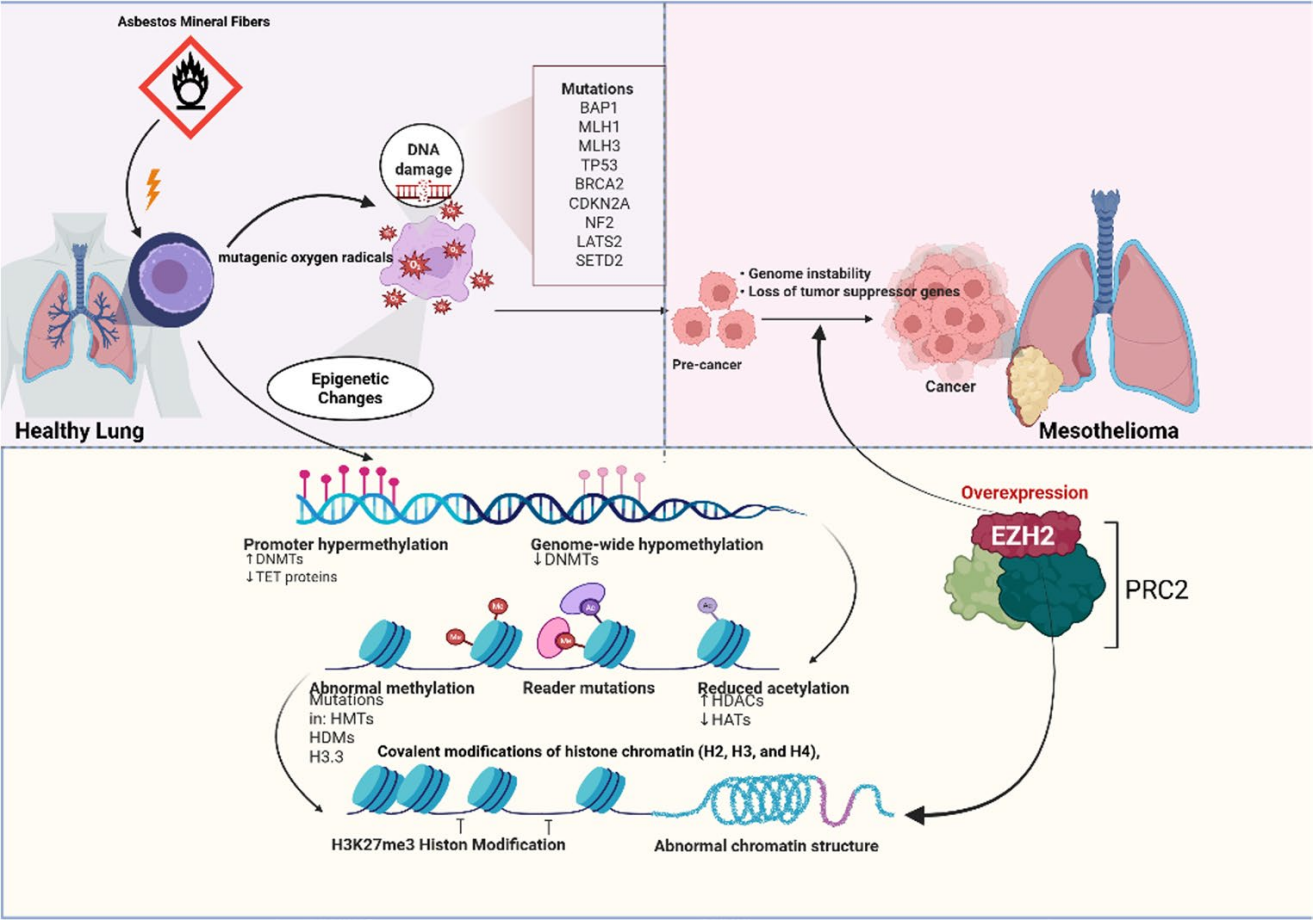
The Pathogenesis of Mesothelioma

Epigenetic mutations associated with histone modifications such as EZH2 and H3K27me3 in addition, mutations in DNA repair genes, particularly BRCA1-Associated Protein 1 (BAP1), are important in explaining the pathogenesis of mesothelioma (Fig. 1). The inflammatory process initiated by asbestos mineral fibers continues as a carcinogenic process with mutations resulting from the emergence of mutagenic oxygen radicals. In addition to these mutations, mutations in DNA repair genes, especially BAP1, play a role in this process [8]. The presence of somatic mutations in BAP1 in approximately 60% of mesothelioma indicates that it is of critical importance [9]. In addition, germline mutations regulating DNA repair such as MLH1, MLH3, TP53, BRCA2 and somatic mutations in CDKN2A, NF2, TP53, LATS2 and SETD2 have been reported [10, 11]. The relatively low mutational burden in mesothelioma suggests that epigenetic changes may be important determinants in this area. The relationship between DNA methylation status and overall survival in epigenetic studies has also increased our focus on this issue [12]. Epigenetic regulation involves acetylation, methylation, ubiquitination, phosphorylation, and covalent modifications of histone chromatin (H2, H3, and H4), the basic unit of which is the nucleosome.

At the epigenetic level, abnormal DNA methylation and histone modifications are an important feature of MPM. A close relationship has also been noted between DNA hypermethylation and inflammation. High DNA methylation frequency in MPM showed a significantly shorter survival rate. These data suggest that accumulation of DNA methylation is a mechanism affecting the progression of this disease [13].

Enhancer of zeste homolog 2 (EZH2) is a chromatin-modifying protein that is the catalytic subunit of polyethylene repressive complex 2 (PRC2). The function of EZH2 is to trimethylate lysine 27 of histone 3 (H3K27me3) via the PRC2 complex. Mutations and increased expression of EZH2 have been observed in many cancers, including lung, breast, prostate, endometrial, and lymphoma. Increased EZH2 expression in these cancers is also associated with poor prognosis [14]. EZH2 generally leads to the silencing of many tumor suppressor genes through its histone methyltransferase activity. Many studies have shown that EZH2 exerts its epigenetic silencing function through epigenetic regulators such as DNA methyltransferases (DNMTs) and histone deacetylases (HDACs). In malignancies such as MPM, EZH2 may contribute against immunotherapy resistance by regulating immune-related pathways. Preclinical studies show promising efficacy of EZH2 inhibitors, especially when combined with immune checkpoint inhibitors. This may inspire combinations with other targeted drugs or chemotherapies. It may open the way for innovative treatment paradigms for aggressive solid tumors such as MPM. In this review, we will discuss the role of EZH2 in MPM, its evidence as a therapeutic target and possible combined treatment approaches with chemotherapy, novel target therapies and immunotherapies.

## EZH2 Signaling in MPM

**Fig. 2:**
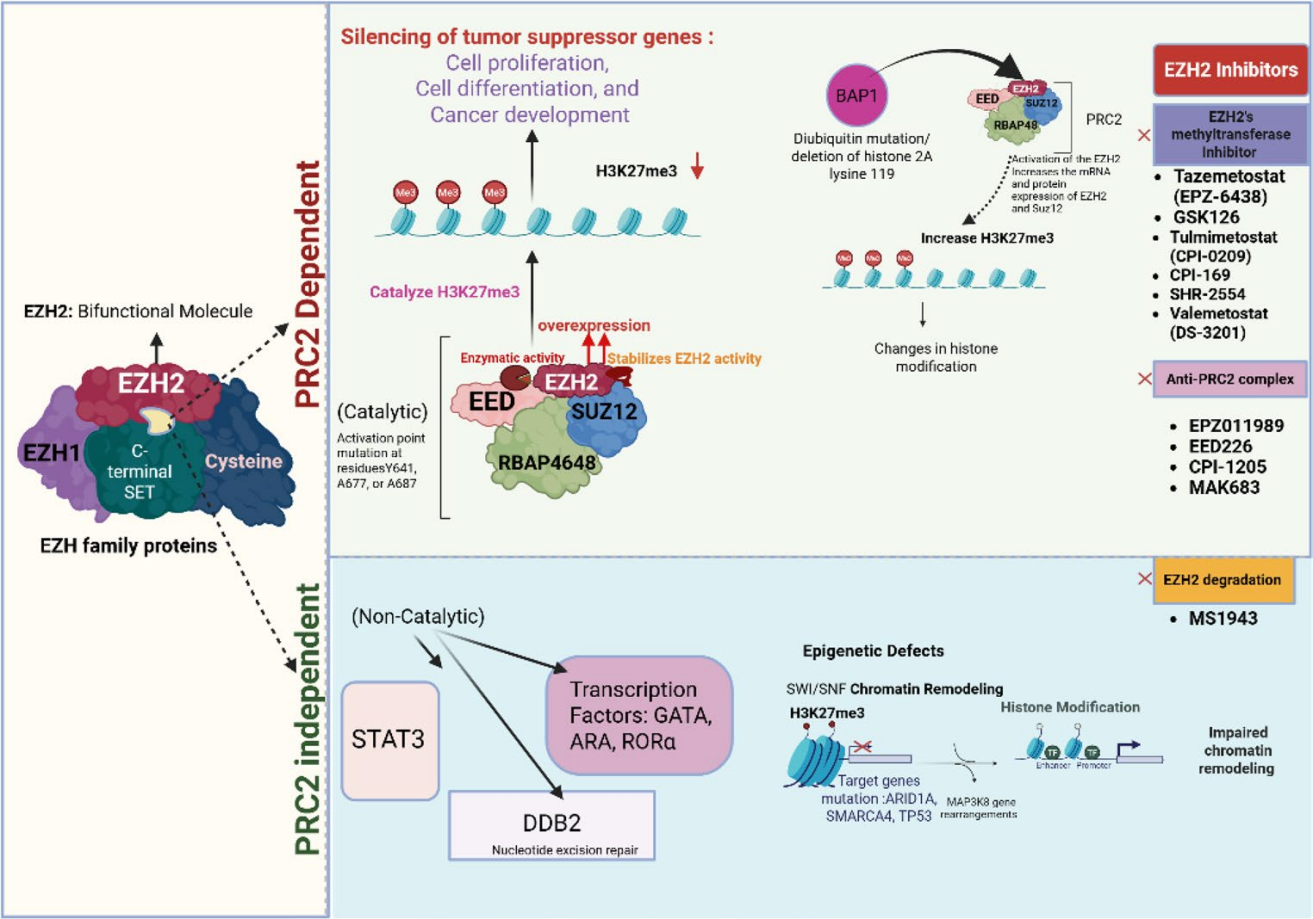
Dual functional roles of EZH2 in Mesothelioma pathogenesis and therapeutic targeting

The EZH family of proteins is comprised of four homologous domains: I (EZH1), II (EZH2), a cysteine-rich domain, and a C-terminal SET domain. The C-terminal SET domain is responsible for the enzyme’s histone methyltransferase activity. EZH2 is one of the major histone methyltransferases and the major catalytic subunit of PRC2, which catalyzes the methylation of histone 3 lysine 27 (H3K27). PRC2 consists of three basic subunits, namely repressor of embryonic ectoderm development (EED), EZH2 and zeste 12 (SUZ12), retinoblastoma (Rb)-associated protein 46/48 (RbAp46/48) and a series of proteins (Fig. 2). The interaction between EZH2 and EED is essential for the enzymatic activity of PRC2. The PRC2 complex methylates the lysine residue at position 27 of histone 3 (H3K27), which promotes chromatin organisation and gene suppression [15]. SUZ12 also stabilizes EZH2 and is required for the HMTase activity and the silencing function of the EED-EZH2 complex. The loss of SUZ12 has been demonstrated to result in the loss of H3K27me3 and the destabilization of EZH2 [16]. In short, when the PRC2 complex is activated and the promoter region of target genes is activated, the SET domain of EZH2 catalyzes H3K27me3, leading the silencing of target genes involved in cell proliferation-differentiation, and cancer development.

EZH2 transcription can occur by both PRC2-dependent (canonical, catalytic) and PRC2-independent (non-canonical, non-catalytic) pathways. This shows that it is a bifunctional molecule. The association of EZH2 with cancer occurs by different mechanisms through these two pathways. The canonical pathway is that EZH2 trimethylates the 27th lysine of histone H3 (H3K27me3), leading to gene silencing. This function increases cell proliferation, particularly by inhibiting the expression of tumour suppressor genes. One of the examples supporting the functional importance of this canonical pathway is the activation mutations at positions Y641, A677 and A687 in the EZH2 gene [17]. These mutations increase the methyltransferase activity of EZH2, raising H3K27me3 levels and potentiating gene silencing [18]. The non-canonical pathway is one in which EZH2 regulates gene activation by interacting with or methylating transcription factors (AR, STAT3, NF-κB, β-catenin) independently of its methyltransferase activity. For example, in hormone-refractory prostate cancer, phosphorylation of EZH2 causes it to methylate the androgen receptor (AR) and this receptor becomes a transcriptional co-activator [19]. This non-PRC2 activity, that is the non-canonical pathway, indicates that EZH2 can play not only a suppressive but also an activating role. This bi-function of EZH2 is of great importance in tumours such as mesothelioma where epigenetic alterations are common. Studies have shown that EZH2 expression is high in mesothelioma and contributes to cell proliferation, invasion and chemotherapy resistance through non-PRC2 pathways as well as PRC2-dependent suppressor gene silencing. In particular, EZH2 inhibitors such as DZNep have been shown to induce apoptosis in mesothelioma cells, suggesting that both catalytic and non-catalytic EZH2 activities should be targeted for treatment.

The canonical pathway of EZH2 methylates lysine 27 on histone 3 (H3K27me3), resulting in gene repression, and EZH2 enzyme inhibitors (GSK126, Tazemetostat) are used in therapies directed against this pathway. However, recent studies have revealed that EZH2 also contributes to cancer progression through a non-canonical pathway. In this pathway, EZH2 activates gene expression by directly interacting with transcription factors (AR, STAT3, MYC) in a PRC2-independent way. These interactions involve novel mechanisms such as transcriptional activation or protein degradation instead of genetic repression. These non-canonical novel functions of EZH2 explain the lack of efficacy of inhibitors of the canonical pathway. As a novel approach, PROTAC drugs (MS-1943), which completely knock down EZH2, may offer more effective therapeutic strategies by affecting both pathways beyond inhibitors of the canonical pathway [20].

Recent findings suggest that in addition to its canonical H3K27me3 methylation function, EZH2 also contributes to oncogenesis by directly interacting with transcription factors or methylating non-histone proteins [21] Some of these non-canonical functions are dependent on methyltransferase activity, while others are completely independent of this activity. For example, EZH2’s methylation of STAT3 increases its DNA binding and expression of target genes, while its interaction with MYC promotes tumor progression by enhancing the transcriptional activity of MYC rather than gene silencing. These mechanisms are outside the classical PRC2 complex and demonstrate that EZH2 can act as a transcriptional activator. Furthermore, EZH2 methylates some proteins, such as RORa, leading to their degradation through protease degradation, resulting in inhibition of cell differentiation and increased proliferation. Another important example is its interaction with DDB2 (DNA damage-binding protein 2). EZH2 methylates DDB2, reducing its stability, which leads to reduced DNA repair capacity and increased genomic instability of tumor cells. These multifaceted interactions demonstrate that EZH2 is not only a gene silencer but also an oncogenic regulatory hub, highlighting the need to go beyond traditional inhibitors in therapeutic targeting.

SWI/SNF, a chromatin remodeling complex, is an important epigenetic mechanism regulating cellular gene expression and mutations in its subunits (ARID1A, SMARCA4) have been detected in many tumor types. In addition to ARID1A and SMARCA4, emerging evidence emphasizes the importance of MAP3K8 gene rearrangements and TP53 mutations. These mutations are thought to be factors contributing to the limited efficacy of EZH2 inhibitors [22, 23]. Therefore, new therapeutic approaches targeting both catalytic and non-catalytic functions of EZH2 were investigated. Cancer cells carrying mutations in SWI/SNF complex genes were found to be dependent on EZH2 not only for its methyltransferase activity, but also for its non-catalytic functions that enable PRC2 stabilization. EZH2 knockdown in these cells does not show the desired effect on cell proliferation with catalytic inhibitors alone. Especially in ARID1A, SMARCA4 and PBRM1 mutated cell lines, tumor development is markedly impaired when the structural integrity of PRC2 is not maintained. Therefore, next-generation therapeutic strategies that target the non-catalytic oncogenic roles of EZH2 beyond classical EZH2 inhibitors may provide a more effective and comprehensive therapeutic response, including in tumors with ARID1A and/or SWI/SNF mutations.

One of the common genetic alterations in MPM is a mutation in the BRCA1-associated protein 1 (BAP1) gene. Loss of BAP1 mutations are associated with changes in histone modification, a critical epigenetic mechanism for gene regulation. BAP1 is an enzyme that functions in the cell nucleus and plays an important role in the regulation of tumour suppressor genes. BAP1 nuclear deubiquitinase targets histones (together with ASXL1 as a Polycomb repressive subunit) and the HCF1 transcriptional cofactor. It has been shown that BAP1 suppression in MPM cell lines affects E2F and Polycomb target genes [24]. As part of the PR-DUB (Polycomb Repressive Deubiquitinase) protein complex, this enzyme binds to lysine at position 119 of histone H2A protein and works through small proteins called ‘monoubiquitin’. This function of BAP1 is to help cells to keep genes that manage vital processes such as differentiation and DNA repair active. Thus, cells can continue to function properly and divide in a controlled way. However, when BAP1 loses its function, this mechanism is impaired and epigenetic suppressor signals that silence genes increase. The effects of BAP1 at the epigenetic level are not only seen as a tumor suppressor gene, but also as a regulator in targeting other therapeutic strategies such as EZH2. Deubiquitin mutation or deletion of histone 2 A lysine 119 in the BAP1 gene, which is common in MPM, causes an increase in H3K27me3 by activation of the EZH2 subunit of the PRC2 complex [25]. In particular, as the PRC2 (Polycomb Repressive Complex 2) epigenetic complex becomes more dominant, it prevents the expression of tumour suppressor genes such as CDKN2A that slow down or stop the cell cycle via H3K27me3 [26]. It has also been shown that Bap1 increases the mRNA and protein expression of EZH2 and SUZ12, which is related to its role in regulating PRC2 expression. With the idea that this increase could be a therapeutic target, the effect of EZH2 inhibition in BAP1 mutant mesothelioma has been investigated [27]. The effect of the EZH2 inhibitor Tazemetostat has been investigated in BAP1 mutant mesothelioma patients with loss of nuclear expression and has shown a high disease control rate [28]. These data suggest that EZH2 inhibitors may work not only in mesothelioma but also in other cancers with BAP mutation.

In conclusion, EZH2 is thought to function as a PRC2-independent transcriptional coactivator in addition to its known role as an H3K27 histone methyltransferase and transcriptional repressor. Further understanding of this bifunctional aspect of EZH2 will be critical in developing personalized treatment approaches and combination therapies to overcome resistance mechanisms.

## EZH2 Inhibitors and Therapeutic/Clinical Evidence in MPM

Epigenetic regulation is a new approach to cancer treatment. We know that epigenetic targeted therapies are particularly effective in hematologic malignancies. However, the approval of Tazemostat, the first EZH2 inhibitor, in a solid cancer type such as Sarcoma was a turning point. Since then, EZH2 inhibitor drugs have been studied in many solid cancer types including MPM (Table 1 [27–39]). In MPM, EZH2 expression was detected at rates as high as 85% compared to normal mesothelial cells. In preclinical studies, pharmacological inhibition of EZH2 with the DZNep significantly suppressed proliferation by reducing H3K27me3 levels in tumor cells. These preclinical data show promise for targeted epigenetic treatment strategies in MPM [40]. EZH2 inhibitors are evaluated in three groups. The first group is the inhibition of EZH2’s methyltransferase activity, which occurs through the competitive effect of S-adenosylmethionine (SAM), an active methyl carrier. This group includes selective ezh2 inhibitors Tazemetostat, GSK126, EPZ011989, and Tulmimetostat(CPI-0209) or dual EZh1 and EZH2 inhibitors Valemetostat. The second group are drugs such as EED226, EED Inhibitors, which act by disrupting the structure of PRC2, of which EZH2 is a subunit. The third group consists of drugs like MS1943 that induce EZH2 degradation [41]. It was thought that EZH2 enzymatic inhibitors or only the PRC2 complex could stop oncogenic activity if they completely suppressed protein interactions. However, since EZH2 has both methylase-dependent and methylase-independent functions, these first drug groups alone may not be sufficient to suppress non-canonical activities. Therefore, new EZH2 inhibitors are being developed to block the catalytic and non-catalytic activities of EZH2 and overcome resistance mechanisms. Advances in drug design offer new opportunities. Proteolysis-targeting chimeras (PROTACs) have emerged as next-generation therapeutic tools, selectively degrading EZH2 proteins rather than merely inhibiting their enzymatic activity. Recently, new drugs such as MS1943 have been developed that target both the catalytic and noncatalytic activities of EZH2 [39]. These new strategies offer a broader therapeutic potential compared to traditional EZH2 inhibitors that primarily target methylation activity. Here, we will focus on preclinical and clinical studies on the essential drugs from these three groups, particularly for the treatment of MPM.

**Table 1:** Completed clinical trials of EZH2 inhibitors in mesothelioma and other solid tumors

**1. EZH2 methyltransferase inhibitors**						
**Tazemetostat**						
NCT Number	Phase	Tumor Types	Drug	Number of patients	Outcomes	Completion/Available Date
NCT02860286 [28]	II	Mesothelioma *BAP1* Loss of Function	Tazemetostat	74	DCR:54% (2 pts PR)	1/5/2019
NCT02601950 [29]	II	Soft Tissue Sarcoma characterized by loss of *INI1/SMARCB1*	Tazemetostat	62	ORR:15% mPFS:5.5mo mOS: 19.0mo	2/26/2024
NCT03213665 [30, 31]	II	Multiple solid tumors	Tazemetostat	20	ORR: %5 (Cohort of children)	12/31/2024
NCT03854474 [32]	I	Urothelial carcinomas	Tazemetostat and pembrolizumab	25	mPFS:3mo PR: Arm A: 30% Arm B:25%	5/29/2024
**GSK2816126 (GSK126)**						
NCT02082977 [33]	I	Multiple solid tumors	GSK2816126	41	MTD SD (34%)	06/20/2017
**Tulmimetostat (CPI-0209)**						
NCT04104776 [34–36]	I/II	*Solid tumors and Lymphoma*	Tulmimetostat	117	Best responses of ≥ 1 CR/PR were seen in 5 cohorts (Lymphoma, Urothelial, Ovarian, Endometrial, Mesothelioma)	5/29/2024 (prelim)
2. **Anti-PCR2 Complex**						
**EPZ011989**						
N/A [27]	Preclinic	Loss of BAP1 MPM cells	EPZ011989	N/A	Sensitive to EPZ011989	8/19/2015
**CPI-1205**						
NCT03480646 [37]	I	mCRPC	CPI-1205 plus enzalutamide or abiraterone/prednisone	25	Well tolerated, with acceptable safety.	7/1/2019
**MAK683**						
NCT02900651 [38]	I/II	CCCO, CRPC, DLBCL, ES, GC, NPC, SWI/SNF-mutated sarcoma	MAK683	139	Clinical activity was observed in patients with advanced DLBCL and ES.	2/5/2025
3. **EZH2 Degradation**						
**MS1943**						
N/A [39]	Preclinic	TNBC	MS1943	N/A	Cytotoxic effect in multiple TNBC cells, while sparing normal cells	12/9/2019

## EZH1/EZH2 Enzymatic Inhibitors

### Tazemetostat

Tazemetostat (EPZ-6438) initially developed by SAM, Tazemetostat is the first class EZH2 inhibitor of its type. It is approved for the treatment of epithelioid sarcoma and follicular lymphoma by FDA and is a milestone in the development of EZH2-targeted therapies [42]. Tazemetostat reactivates silenced tumor suppressor genes by reducing H3K27me3 levels and stops tumor progression. Tazemetostat was evaluated in Phase II clinical trials specifically for the treatment of patients with BAP1-inactivated MPM. This study included 74 patients that selected patients had BAP1-inactivated tumors [28]. In the BAP1-inactivated tumor cohort, the disease control rate was 54%, whereas in the other cohort was 51%. Median progression-free survival and overall survival were similar in both the BAP1-inactivated group and the overall group. Tazemetostat had good tolerability. Most of adverse events were fatigue, decreased appetite and nausea. This data shows that Tazemetostat had an acceptable safety profile alongside some clinical benefits for relapsed or refractor MPM patients, including those with BAP1 inactivated tumors. Phase 2 results indicate some therapeutic advantages and support further research, especially in combination with other agents to increase efficacy. There are currently no phase III clinical trials specifically evaluating Tazemetostat in mesothelioma. Randomized phase III studies are needed to determine efficacy and safety.

### Valemetostat

Unlike the first-generation Tazemetostat group of drugs, Valemetostat has a dual inhibitory property that targets both EZH1 and EZH2. In this respect, it provides a more comprehensive effect. Studies have focused on its use in treating hematological tumors, such as non-Hodgkin lymphoma and adult T-cell leukemia/lymphoma. In a phase 2 study of patients with relapsed or refractory peripheral T-cell lymphoma (PTCL), it demonstrated a significant objective clinical response rate of 44%, a remarkable result compared to current treatments for this treatment-resistant patient group [43]. The drug’s safety profile was generally manageable, with the most common adverse events being grade 3–4 thrombocytopenia (23%), anemia (19%), and neutropenia (17%). Clinical studies are ongoing in common solid tumors such as lung cancer (Table 2). Although it has not yet been directly studied on rare cancers such as MPM, it seems to be a promising drug.

**Table 2:** Ongoing clinical trials of EZH2 inhibitors in mesothelioma and other solid tumors

NCT number	Phase	Study name	Drug	Tumor types	Primary Outcomes	Status
NCT04104776	I/II	A Study of Tulmimetostat DZR123 (CPI-0209) in Patients with Advanced Solid Tumors and Lymphomas	Tulmimetostat	Lymphoma and Solid tumors, including Mesothelioma	MTD and RP2D	Recruiting
NCT05467748	I/II	EZH2 Inhibitor, Tulmimetostat, and PD-1 Blockade for Treatment of Advanced Non-small Cell Lung Cancer	Tulmimetostat Plus Pembrolizumab	NSCLC	Safety/tolerability and ORR	Not yet Recruiting
NCT02875548	I/II	A Study to Assess Long-term Safety of Tazemetostat in Adult Participants of All Ages with Any Disease Treated with Tazemetostat in a Previous Clinical Study (TRuST)	Tazemetostat	Lymphoma and Solid tumors, including Mesothelioma	Long-term safety	Active, not recruiting
NCT05023655	II	Phase II Study of Tazemetostat in Solid Tumors Harboring an ARID1A Mutation	Tazemetostat	Solid Tumor with ARID1A Mutation	ORR	Recruiting
NCT04204941	III	Tazemetostat in Combination with Doxorubicin as Frontline Therapy for Advanced Epithelioid Sarcoma	Tazemetostat Doxorubicin HCl	Advanced Soft-Tissue or Epithelioid Sarcoma	DLT PFS	Recruiting
NCT05407441	I/II	Tazemetostat+Nivo/​Ipi in INI1-Neg/​SMARCA4-Def Tumors	Tazemetostat Nivolumab Ipilimumab	Atypical Teratoid Rhabdoid Tumor INI1 (SMARCB1)-deficient Primary CNS Malignant Tumors SMARCA4-deficient Primary CNS Malignant Tumors Malignant Rhabdoid Tumor (MRT) Rhabdoid Tumor of the Kidney (RTK) Epithelioid Sarcoma Chordoma	MTD and Safety	Recruiting
NCT05353439	I	Testing of Tazemetostat in Combination with Topotecan and Pembrolizumab in Patients with Recurrent Small Cell Lung Cancer	Pembrolizumab, Tazemetostat Topotecan	SCLC	MTD	Recruiting
NCT04557956	I/II	Testing the Addition of the Anti-cancer Drug, Tazemetostat, to the Usual Treatment (Dabrafenib and Trametinib) for Metastatic Melanoma.	Dabrafenib Trametinib, Tazemetostat	Melanoma	RP2D mPFS	Recruiting
NCT06807632	I	A Study of Valemetostat in Combination with Atezolizumab in People with Lung Cancer	Valemetostat, Atezolizumab	AS-SCLC	RP2D	Recruiting
NCT06294548	I/II	A Study of Valemetostat Tosylate (DS-3201b) With Atezolizumab and Bevacizumab in HCC	Valemetostat, Atezolizumab, Bevacizumab	HCC	MTD/RP2D ORR	Recruiting
NCT06644768	I/II	A Study of Valemetostat Tosylate Plus Pembrolizumab Versus Pembrolizumab Alone in First-Line NSCLC Without Actionable Genomic Alterations	Valemetostat, Pembrolizumab	NSCLC	DLT, PFS	Recruiting
NCT05633979	I	Phase 1b Study of EZH1/​2 Inhibitor Valemetostat in Combination with Trastuzumab Deruxtecan in Subjects with HER2 Low/​Ultra-low/​Null Metastatic Breast Cancer	Trastuzumab deruxtecan, Valemetostat	Breast	MTD/RP2D ORR	Recruiting
NCT05879484	I/II	Study of Front Line Pembrolizumab and Valemetostat in PD-L1 Positive, HPV-Negative Recurrent/​Metastatic Squamous Cell Carcinoma (SCC) of the Head and Neck: The PANTHERAS	Pembrolizumab, Valemetostat	NSCLC-SCC, HNSCC	RP2D	Recruiting

### GSK126

Another EZH2 inhibitor is GSK126, which demonstrates impressive activity in both clinical and preclinical studies. In phase I trials, patients with EZH2 mutations exhibited partial responses in 40% of cases, with disease stabilization occurring in a further 35% of cases, which is promising [33]. This is also likely to be useful in other tumor types, especially MPM. In preclinical studies, GSK126 has been evaluated for its potential therapeutic effects in MPM. It was demonstrated that GSK126 decreased cell viability and apoptosis in BAP1-deficient MPM cell lines. They have examined the use of GSK126 with other treatment modalities. The results in particular are remarkable with GSK126 and FGFR inhibitors in BAP1-deficient MPM models. GSK126 was able to reduce the tumor volume substantially in BAP1 mutant mice, and with the added benefit of FGFR inhibitors, tumor growth was significantly stunted (*p* < 0.05), along with survival rates enhancing to almost two-fold [26]. These results warrant further clinical studies involving dual EZH2/FGFR blockade as a prospective treatment plan for mesothelioma. The other study found that the combination of the EZH2 inhibitor GSK126 and the ATM inhibitor AZD1390 reduced tumor volume by approximately 60% in BAP1-mutant mesothelioma tumors. This effect was statistically significant (*p* = 0.01). Additionally, a decrease in H3K27me3 levels was observed in tumor tissues of mice treated with this combination, indicating activation of the DNA damage response and elimination of epigenetic methylation. These results suggest a clear biological response to the treatment, indicating that this strategy could be effective for treating BAP1-mutant MPM [44]. Another studies have investigated the relationship between GSK126 and the tumor immune microenvironment. GSK126 treatment was characterized by an increase in myeloid-derived suppressor cells (MDSCs) and a decrease in the infiltration of CD4 and CD8 T cells, which are critical for antitumor immunity [45]. MDSCs suppress the immune response of immune cells against the tumor, thereby reducing the effect of the immune system. Apart from the direct antitumor effects of GSK126, it also promotes the expansion of MDSCs and therefore may synergize with agents that activate the tumor immune system. Combining GSK126 with agents that deplete MDSCs or increase T cell activity could potentially improve therapeutic outcomes.

### EPZ011989

EPZ011989 is a second-generation EZH2 inhibitor with high selectivity and efficacy that was evaluated in preclinical studies for its potential therapeutic effects in the treatment of malignant pleural mesothelioma (MPM) [27]. Preclinical studies showed that treatment with EPZ011989 reduced tumour size by 60–70% in BAP1-mutant tumors. Treatment with EPZ011989 significantly reduced tumor growth, invasion and pulmonary metastasis by approximately 50% in BAP1-mutant mesothelioma models. Treatment with EPZ011989 at a concentration of 1.25 µM for two weeks reduced cell viability by over 50% and significantly increased apoptosis (*p* < 0.005). Similarly, when tested in 3D culture media, the drug was reported to suppress cell proliferation by up to 60%. In tumor models, EPZ011989 showed higher efficacy in mutant cell lines compared to wild-type BAP1 cell lines and reduced invasion by increasing E-cadherin expression. This suggests that EPZ011989 may have therapeutic potential in the treatment of MPM, particularly in cases involving BAP1 mutations. It has been shown that BAP1-mutant malignant pleural mesothelioma (MPM) cells are sensitive to the selective EZH2 inhibitor EPZ011989. In contrast, treatment sensitivity has been limited in BAP1 wild-type tumors. One reason for this is that EZH2 inhibition has been reported to reduce proliferation in BAP1 wild-type cells under conditions of low SIRT1 levels. Since CDKN2A expression status may influence the response to EZH2 inhibitors, it is recommended that patients be evaluated based on their CDKN2A status in a translational context [46]. In another preclinical study, EPZ011989 strongly inhibited both mutant and wild-type EZH2 and significantly suppressed cell proliferation in lymphoma cells. When administered twice daily at doses of 250 and 500 mg/kg in a 21-day in vivo DLBCL xenograft model, it significantly suppressed tumor growth and completely eliminated histone H3K27me3 methylation at the 500 mg/kg [47]. There are currently no clinical trials evaluating EPZ011989 in mesothelioma patients. Further research is needed to assess the efficacy and safety of EPZ011989 both as monotherapy and in combination with other treatments.

### Tulmimetostat (CPI-0209)

Tulmimetostat a second-generation epigenetic inhibitor targeting EZH1 along with EZH2, provides more sustained and comprehensive PRC2 suppression than first-generation EZH2 inhibitors. It has shown potent antitumor effects in tumors carrying mutations in epigenetic regulatory genes such as ARID1A or BAP1. In the Phase 1/2 study NCT04104776, tulmimetostat was evaluated in six different cohorts, one of which was a patient group with BAP1 loss of pleural mesothelioma [48]. Of the 21 patients evaluated in this cohort, 14% achieved a partial response (PR) and 48% had stable disease (SD), increasing the overall disease control rate to 62%. Tulmimetostat’s efficacy in this patient group suggests that EZH1/2 dual inhibition may be considered as a treatment strategy in tumors with high epigenetic dependence such as MPM.

## EZH2 Degrader (MS1943)

MS1943 is a next-generation EZH2 targeting agent that causes proteasomal degradation of EZH2. The development of selective EZH2 degraders relies on the use of proteolytic targeting chimeras (PROTACs) which aim to enhance inhibition of EZH2 through specific binding using hydrophobic tagging [49]. What makes MS1943 unique is that it is a potent degrader that uses a non-covalent inhibitor to bind to the target protein. Studies have shown that MS1943 significantly reduces cell proliferation as monotherapy. MS1943 reduced the levels of EZH2 protein in several cancer cell lines, including the prostate cancer cell line. This was achieved without any effect on other members of PRC2 such as EZH1, SUZ12, and EED. This demonstrates the high selectivity of MS1943 in PRC2 complex-dependent targeting strategies. EZH2 is overexpressed in a wide variety of cancers, including triple negative breast cancer (TNBC), where its elevated expression correlates with poor clinical outcomes. Administration of MS1943 at 150 mg/kg/day resulted in marked inhibition of tumor cell growth and H3K27me3 reduction in TNBC models, both in vitro and in vivo (*p* = 0.03) [39]. MS1943 induces apoptosis in TNBC cells by directly eliminating EZH2 through the PROTAC mechanism, leading to increased caspase-3 levels (*p* = 0.01). Unlike classical inhibitors, the complete elimination of the EZH2 protein has produced stronger biological responses. MS1943 has not yet progressed to preclinical clinical phases in MPM, but based on preclinical findingsin other cancers, it has great potential, especially for tumors in which EZH2 is overexpressed. Because this agent completely eliminates the target protein, unlike current EZH2 inhibitors, it is thought to be a more durable and effective treatment against resistance.

## Destabilizers of the PRC2 Complex (EED Inhibitors: EED226)

EED226 is a potent inhibitor of the EED protein, a critical subunit of the PRC2. EED226 binds to the H3K27me3 pocket in the EED subunit, destabilizing the PRC2 complex and leading to the inhibition of EZH2 function. EED226 suppresses H3K27 trimethylation, leading to the re-activation of tumor suppressor genes. Although EED226 was found to possess good tumor treatment, EZH2 selectivity, and bioavailability, issues still remain in modeling solubility and stability. Researchers maximized the structure of EED226 and developed MAK683 [50]. EED226 has been found in preclinical models to reduce levels of H3K27me3 [51]. While the EZH2 inhibitor Tazemetostat, currently in clinical use, has limited efficacy, EED226, which directly inhibits EED, offers a potentially more effective approach to suppressing the full function of PRC2. Preclinical studies are needed to evaluate the effects of EED226 on MPM. Such studies will help us better understand the potential of EED226 in the treatment of MPM.

In conclusion, EZH2 enzymatic inhibitors, starting with Tazemetostat and including new ones such as GSK126, EPZ011989, MS1943 and EED226, could be important tools in the fight against MPM. Indeed, high rates of response proved in preclinical studies can potentially have potential in the treatment of MPM with regression of the tumor. In contrast to drugs that inhibit the methyltransferase activity of EZH2, drugs that target the PRC2 complex, such as EED and SUZ12, or EZH2 degraders, have been developed. EED and SUZ12 play critical roles in stabilizing and maintaining the function of the PRC2 complex, making them targets for disruption of EZH2 activity. In addition to EED226 and MS1943, other research efforts have focused on targeting other PRC2 components such as SUZ12 and RbAp46/48. Small molecule and peptide-based inhibitors are being developed to interfere with these components and potentially disrupt the entire PRC2 complex. These approaches aim to initiate epigenetic reprogramming and further advance the sensitization of tumor cells to new therapy agents. Preclinical and clinical tests of MS1943 and EED226 drugs are needed in cancer that is associated with PRC2 activity, especially in MPM.

## EZH2 Inhibitors and Immunotherapy and Future Perspectives

**Fig. 3:**
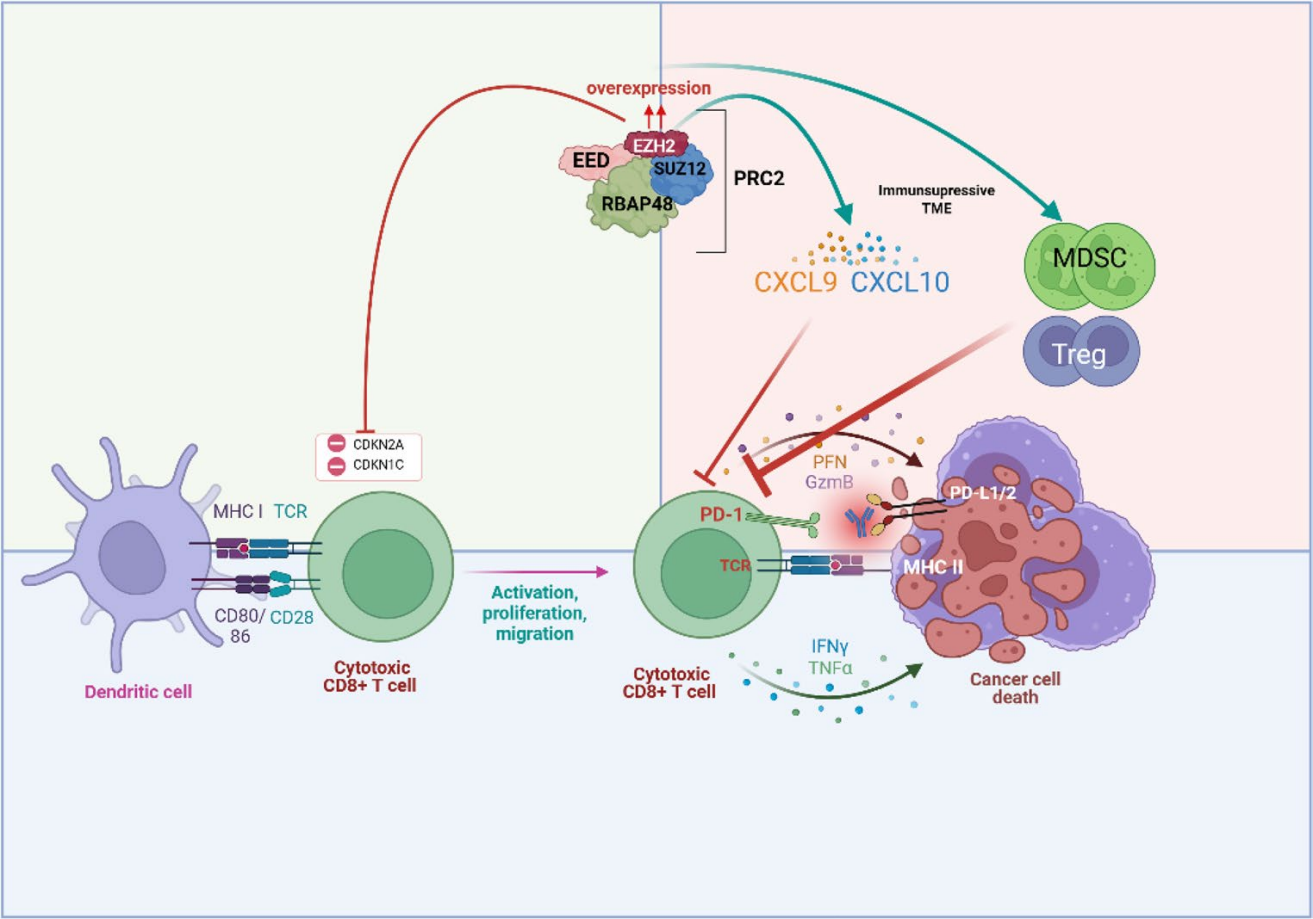
EZH2-mediated immune evasion and modulation of the tumor microenvironment in Mesothelioma

Immune checkpoint inhibitor therapies have been approved as a new treatment other than platinum-based chemotherapy treatments in the treatment of mesothelioma for the first time after a long time. However, resistance to immunotherapy is an important factor limiting the effectiveness of treatment. The molecular mechanisms of resistance to immunotherapy are still being investigated. Epigenetic changes have been associated with resistance to immunotherapy (Fig. 3). Induction of the histone methyltransferase Ezh2 has been shown to control various resistance mechanisms. The relationship between EZH2 gene expression and immune cell infiltration in the tumor microenvironment has been investigated in some cancers [52]. A positive correlation has been shown between CD8 + T cells, regulatory T cells (Tregs), macrophages and EZH2 infiltration. CD8 + T cells play a important role in antitumor immunity in cancer. EZH2 is expressed to varying degrees on CD8 + T cells at different stages. EZH2 promotes CD8 + T cell proliferation by inhibiting the expression of the cell cycle regulators cyclin-dependent kinase inhibitor CDKN2A and CDKN1C. In these studies, during anti-CTLA-4 or IL-2 immunotherapy, intratumoral tumor necrosis factor-α (TNF-α) production and T cell accumulation led to increased Ezh2 expression in tumor cells, which silenced tumor immunogenicity and antigen presentation. These antitumor effects were dependent on the accumulation of intratumoral interferon-g (IFN-g)-producing Programmed Cell Death Protein (PD-1) low CD8 + T cells and downregulation of PD-L1 in tumor cells. Ezh2 inactivation reversed this resistance and synergized with anti-CTLA-4 and IL-2 immunotherapy. Therefore, Ezh2 acts as a molecular switch controlling tumor escape during T cell-targeted immunotherapies. Thus, epigenetic modulation of EZH2 is not limited to oncogenesis but also profoundly affects immune evasion mechanisms. Based on this information, clinical trials of EZH2 inhibitors in combination with not only checkpoint inhibitors but also chemotherapy are continuing to be investigated in various cancers (Table 2). EZH2 creates an immunosuppressive tumor microenvironment by suppressing the expression of important antigen presentation molecules, including CXCL9 and CXCL10. These molecules are required for the recruitment of CD8 + T cells, and their downregulation leads to decreased immune control [53, 54]. Furthermore, tumors with high EZH2 expression promote the increase of immunosuppressive populations such as Tregs and MDSCs. The combination of EZH2 inhibition and immunotherapy may overcome resistance mechanisms due to the direct effect of EZH2 on tumor growth and its role in indirectly regulating the immune response. The positive preclinical results will encourage the initiation of clinical trials.

The mutational burden of MPM, particularly BAP1, is closely associated with immune evasion and tumor progression. BAP1 mutations affect the tumor microenvironment by suppressing the recruitment of CD8 + T cells, a critical component of antitumor immunity, through molecules such as CXCL9 and CXCL10 [27]. In addition, cancers with these mutations have an increased prevalence of immunosuppressive cell groups such as Tregs and myeloid-derived suppressor cells (MDSCs). For example, preclinical studies have shown that BAP1-deficient MPM tumors exhibit a significant reduction in cytotoxic T cell infiltration, which is associated with poor outcome. BAP1 mutations have been associated with increased expression of EZH2, an epigenetic regulator that contributes to tumor immune evasion by silencing tumor suppressor genes and reducing antigen presentation capacity. Understanding these interactions is critical to designing therapeutic approaches that effectively target both epigenetic and immune dysfunctions. Apart from BAP1 mutation, recent studies have highlighted the interaction of epigenetic regulators such as F6A and KDM6B, which act as demethylases to neutralize the methylating activity of EZH2, with the immune microenvironment. Dysregulation of the balance of activities of these proteins leads to uncontrolled H3K27 methylation, impairing normal cell differentiation and promoting an immunosuppressive tumor microenvironment in MPM. For example, KDM6A mutations, which are common in MPM, disrupt the demethylating function, further tipping the balance in favor of EZH2-mediated silencing of tumor suppressor genes. This imbalance not only drives tumor growth but also suppresses the production of chemokines required for T cell recruitment, promoting immune evasion. The integration of EZH2 inhibitors with immunotherapy offers a promising synergistic approach for the treatment of MPM. EZH2 inhibition not only improves antigen presentation but also remodels the tumor microenvironment (TME). This remodelling enhances the mobilization and activation of effector T cells and memory chimeric antigen receptor (CAR) CD8 + T phenotypes, while reducing immunosuppressive elements such as Tregs and MDSCs [53]. It has also been suggested that EZH2 inhibition may enhance the function of dendritic cells within the TME, thereby further enhancing the anti-tumor immune response. Preclinical studies have shown that EZH2 inhibitors enhance the efficacy of immune checkpoint inhibitors (ICIs), even in tumours that are highly resistant to monotherapy [55]. Furthermore, the combination of EZH2 inhibition with ICIs offers the potential to transform “cold tumours” lacking sufficient immune infiltration into “hot tumours” by promoting T-cell recruitment. Immunotherapy is changing the treatment paradigm for MPM, the integration of novel targets such as EZH2 has the potential to further improve patient outcomes. The investigation of such combinations highlights the importance of a multifaceted approach to this challenging malignancy. Combining EZH2 inhibitors with immune checkpoint inhibitors has shown potential to reverse immune suppression and restore antitumor immunity, particularly in BAP1 mutant tumors. Such combination therapies may not only reduce tumor growth but also enhance the efficacy of immune-based therapies, enabling the development of personalized strategies for MPM.

Epigenetic mechanisms play an important role in cancer development and in recent years EZH2 and its role in the epigenetic process has been investigated. Targeted therapies have shown significant success in some cancers, particularly lung adenocarcinoma, but no such targeted therapy has yet been shown in mesothelioma. One of the main reasons for this is the dominant role of tumor suppressor genes rather than oncogenic activating genes in mesothelioma. For this reason, targeting tumor suppressor genes such as NF2, BAP1 and PIK3CA has become a subject of research. As with all targeted therapies, drug resistance is a major challenge for EZH2 inhibitors. EZH2 inhibition may activate alternative oncogenic pathways, such as PI3K/Akt or MEK/ERK signaling. Combination regimens with other target drugs can be used in this strategy to overcome resistance in EZH2-targeted therapies. Combining EZH2 inhibitors with targeted therapies, such as PI3K, MEK, or Akt inhibitors, has shown potential to address resistance mechanisms. There is not yet an approved drug that directly targets the BAP1 mutation in mesothelioma treatment. BAP1 is a tumor suppressor involved in DNA repair. Tumor cells with BAP1 mutation may be sensitive to PARP group inhibitors, another group involved in DNA repair. Clinical study with single-agent PARP inhibitors in MPM have shown partial efficacy [56]. Therefore, the combination of PARP inhibitors with EZH2 inhibitors may be a potential therapeutic strategy to be evaluated especially in BAP1-mutant mesothelioma subtypes.

Resistance to EZH2 inhibitors is associated with different mechanisms. Mutations in the SET domain of EZH2 prevent drug binding. When the RB1/E2F pathway that controls the cell cycle is broken, cells continue to divide despite EZH2 inhibition. The BAF complex is involved in the removal of the PRC2 complex from DNA. When this complex (SWI/SNF-dependent chromatin remodeling complex) loses its function, suppressed tumor suppressor genes cannot be reactivated [57]. These resistance mechanisms may limit the use of EZH2 inhibitors alone and create the need for combination therapies.

EZH2 contributes to chemotherapy resistance through both canonical and non-canonical pathways. Canonically, it suppresses the expression of tumor suppressor genes regulating DNA damage response, apoptosis and cell cycle through H3K27 trimethylation within the PRC2 complex. This suppression reduces the sensitivity of cells to chemotherapy. In the non-canonical mechanism, EZH2 supports cell proliferation and survival by methylating or complexing with transcription factors such as STAT3 and β-catenin [58]. EZH2 inhibitors can overcome this resistance and sensitize tumor cells to chemotherapy. For example, studies suggest that EZH2 inhibitors may improve sensitivity in aggressive cancers such as triple-negative breast cancer and lung cancer when used in combination with chemotherapy or immune checkpoint inhibitor drugs, and ongoing studies are available (Table 2). Therefore, it will be necessary to investigate whether EZH2 inhibitors work in combination with pemetrexed and cisplatin chemotherapy. Especially in resistant tumor types such as mesothelioma, these combinations may be an option.

## Conclusion

EZH2 is an important transcriptional regulator, epigenetic and immunological modulator that plays a broad role in cancer development. Although Ezh2-specific clinical studies in mesothelioma are limited, results from preclinical studies are promising. Furthermore, the development of next-generation EZH2 inhibitors and their integration into immunotherapy, targeted therapy or chemotherapy regimens may have the potential to overcome current therapeutic challenges and improve survival in mesothelioma patients.

## Key References

- Cao R, Wang L, Wang H, Xia L, Erdjument-Bromage H, Tempst P, et al. Role of histone H3 lysine 27 methylation in Polycomb-group silencing. Science. 2002 Nov 1;298(5595):1039–43.
  - ○ Of outstanding importance: By identifying the EED–EZH2 complex as the enzymatic driver of H3K27 methylation, this work established the molecular basis for PRC2 function and laid the groundwork for decades of research on chromatin regulation and cancer epigenetics.
- Gan L, Yang Y, Li Q, Feng Y, Liu T, Guo W. Epigenetic regulation of cancer progression by EZH2: from biological insights to therapeutic potential. Biomark Res. 2018;6:10.
  - ○ This review provides a comprehensive synthesis of the multifaceted roles of EZH2 in cancer biology, bridging its epigenetic regulatory mechanisms with tumor progression, immune modulation, and therapeutic targeting.
- McCabe MT, Ott HM, Ganji G, Korenchuk S, Thompson C, Van Aller GS, et al. EZH2 inhibition as a therapeutic strategy for lymphoma with EZH2-activating mutations. Nature. 2012 Dec 6;492(7427):108–12.
  - ○ This landmark study provides the first preclinical evidence that selective inhibition of mutant EZH2 can reverse aberrant H3K27 methylation and suppress lymphoma growth, establishing EZH2 as a viable therapeutic target.
- Zauderer MG, Szlosarek PW, Le Moulec S, Popat S, Taylor P, Planchard D, et al. EZH2 inhibitor tazemetostat in patients with relapsed or refractory, BAP1-inactivated malignant pleural mesothelioma: a multicentre, open-label, phase 2 study. Lancet Oncol. 2022 Jun;23(6):758–67.
  - ○ This clinical trial represents one of the first evaluations of EZH2 inhibition in solid tumours, demonstrating meaningful disease control in patients with BAP1-inactivated malignant pleural mesothelioma and establishing a translational bridge from epigenetic biology to therapeutic application. By highlighting the biomarker-driven efficacy of tazemetostat in a hard-to-treat cancer, this study underscores the clinical potential of targeted epigenetic therapies and paves the way for refined patient selection strategies.
- Kazansky Y, Cameron D, Mueller HS, Demarest P, Zaffaroni N, Arrighetti N, et al. Overcoming clinical resistance to EZH2 inhibition using rational epigenetic combination therapy. Cancer Discovery. 2024 Jun 1;14(6):965–81.
  - ○ By identifying rational combination strategies to overcome tazemetostat resistance, this work establishes a forward-looking framework for durable epigenetic cancer therapy and informs future clinical trial design.

## Data Availability

No datasets were generated or analysed during the current study.

## Abbreviations

EZH2: Enhancer of Zeste Homolog 2
H3K27me3: Trimethylation of Lysine 27 on Histone H3
PRC2: Polycomb Repressive Complex 2
BAP1: BRCA1-Associated Protein 1
DNMTs: DNA Methyltransferases
HDACs: Histone Deacetylases
EED: Embryonic Ectoderm Development
SUZ12: Zeste 12
RbAp46/48: Retinoblastoma-Associated Protein 46/48
HMTase: Histone Methyltransferase
SAM: S-Adenosylmethionine
PROTACs: Proteolysis-Targeting Chimeras
MDSCs: Myeloid-Derived Suppressor Cells
CXCL9/10: Chemokine (C-X-C Motif) Ligand 9/10
KDM6A/B: Lysine Demethylase 6 A/B
PI3K/Akt: Phosphoinositide 3-Kinase/Protein Kinase B
MEK/ERK: Mitogen-Activated Protein Kinase Kinase/Extracellular Signal-Regulated Kinase
PARP: Poly (ADP-Ribose) Polymerase
SWI/SNF: SWItch/Sucrose Non-Fermentable (chromatin remodeling complex)
ARID1A: AT-Rich Interaction Domain 1 A
SMARCA4: SWI/SNF-Related, Matrix-Associated, Actin-Dependent Regulator of Chromatin, Subfamily A, Member 4
FGFR: Fibroblast Growth Factor Receptor
DDB2: DNA Damage-Binding Protein 2
STAT3: Signal Transducer and Activator of Transcription 3
NF: κB-Nuclear Factor Kappa-Light-Chain-Enhancer of Activated B Cells
MYC: MYC Proto-Oncogene
RORa: Retinoic Acid Receptor-Related Orphan Receptor Alpha
CDKN2A: Cyclin-Dependent Kinase Inhibitor 2 A
PR: DUB-Polycomb Repressive Deubiquitinase
SET: Suppressor of Variegation, Enhancer of Zeste, Trithorax (domain)
CTLA: 4-Cytotoxic T-Lymphocyte-Associated Protein 4
DZNep − 3: Deazaneplanocin A
LATS2: Large Tumor Suppressor Kinase 2
MLH1/3: MutL Homolog 1/3
TP53: Tumor Protein 53
BRCA2: Breast Cancer Type 2 Susceptibility Protein
NF2: Neurofibromin 2
MAP3K8: Mitogen-Activated Protein Kinase Kinase Kinase 8
PIK3CA: Phosphatidylinositol-4,5-Bisphosphate 3-Kinase Catalytic Subunit Alpha
E2F: E2F Transcription Factor
